# Anti-inflammatory potential of quercetin: From chemistry and mechanistic insight to nanoformulations

**DOI:** 10.1016/j.crphar.2025.100217

**Published:** 2025-03-18

**Authors:** Diwakar Aggarwal, Mayank Chaudhary, Sachin Kumar Mandotra, Hardeep Singh Tuli, Ritu Chauhan, Naveen Chandra Joshi, Damandeep Kaur, Laurent Dufossé, Abhishek Chauhan

**Affiliations:** a Department of Bio-Sciences and Technology, Maharishi Markandeshwar Engineering College, Maharishi Markandeshwar (Deemed to be University), Mullana-Ambala, 133207, Haryana, India; b Department of Biotechnology, Graphic Era (Deemed to be University), Dehradun, 248002, Uttarakhand, India; c Amity Institute of Microbial Technology, Amity University, Noida, 201313, India; d University Center for Research & Development (UCRD), Chandigarh University, Gharuan, Mohali, Punjab, India; e Chemistry and Biotechnology of Natural Products, CHEMBIOPRO, Université de La Réunion, ESIROI Agroalimentaire, 15 Avenue René Cassin, CS 92003, CEDEX 9, F-97744, Saint-Denis, France; f Amity Institute of Environmental Toxicology Safety and Management, Amity University, Noida, U.P., India

**Keywords:** Flavonoids, Inflammation, Metabolic syndromes, Nanoparticles, Quercetin

## Abstract

Flavonoids are hydroxylated polyphenols that are abundantly produced by plants as secondary metabolites. These flavonoids hold vast therapeutic potential as they possess numerous medicinal benefits encompassing anti-inflammatory, anti-oxidative, anticancer and antiviral properties. Flavonoids render anti-inflammatory effect either by activating antioxidant pathways or by inhibiting enzymatic secretions involved in inflammatory reactions. Flavonoids like quercetin targets inflammation by modulating expression of cytokines and pro-inflammatory molecules and by inhibiting pro-inflammatory enzymes. Mode of action, absorption and bioavailability of flavonoids greatly affect their biological activity. On-going research is focussing on isolation, synthesis of flavonoid analogs and effect of flavonoids on human health by manifestation of different techniques and animal models. Unravelling the anti-inflammatory potential of flavonoids can manifest better treatment options against variety of diseases and metabolic syndromes. Additionally, enhanced bioavailability of flavonoids can result in superior pharmaceutical activities.

## Highlights

• Flavonoids exhibit potent anti-inflammatory, antioxidative, anticancer, and antiviral properties.
• Quercetin modulates inflammation by regulating cytokines, proinflammatory molecules, and key enzymes.
• Enhancing flavonoid bioavailability can improve their therapeutic efficacy and pharmaceutical applications.

## Graphical abstract

**Figure:**
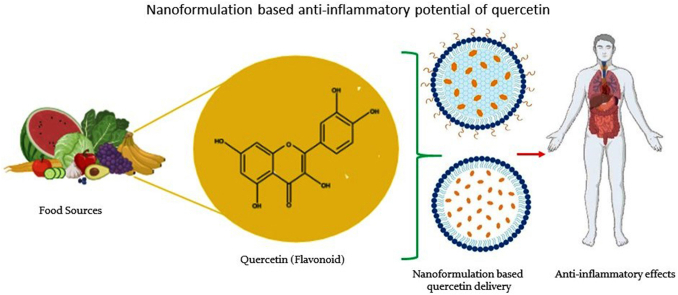

## 1 Introduction

Flavonoids can be classified as bio-flavonoids, iso-flavonoids and neo-flavonoids on the basis of degree of unsaturation, attachment of B ring to C ring through carbon, degree of hydroxylation and oxidation and certain other substitutions (Santos et al., 2017; Ramesh et al., 2021). Flavonoids in which B ring is attached to the C ring at 2nd position are termed as bio-flavonoids and these can further be classified as flavonols, flavones, flavonones, anthocyanidins and chalcones depending upon the structure of C ring (Panche et al., 2016). Most commonly found bio-flavonoids belong to the flavonols subclass and quercetin is one of the most abundant dietetic flavonoid (Hossain et al., 2016) found in fruits and vegetables. Quercetin is a lipophilic compound that is absorbed by simple diffusion and involves both oral and intestinal bacteria for its enzymatic hydrolysis (Cui et al., 2022). Quercetin displays multiple therapeutic roles (antioxidant, anti-inflammatory, anti-apoptotic, anti-aging) through complex mechanisms by intertwining multiple signaling pathways (Chen et al., 2018; Cui et al., 2022).

Anti-inflammatory role of quercetin has been studied in a variety of diseases (Chen et al., 2018; Al-Khayri et al., 2022). Quercetin can inhibit Toll-like Receptor 4 (TLR4) arbitrated expression of inflammatory mediators and cytokines by inhibiting activation of TLR4 (Chen et al., 2018). It can further repress the heightened expression of adhesion molecules and chemokines (Bhaskar et al., 2016). LPS-induced inflammation is prevented by quercetin by inhibiting Src and Syk-mediated P13K phosphorylation (Yang et al., 2014). Neurodegenerative diseases related inflammatory responses include activation of glial cells and up-regulation of free radicals and inflammatory markers. Studies have reported anti-inflammatory activity of quercetin against neuronal diseases (Spagnuolo et al., 2018). Quercetin reduced neuroinflammation in Parkinson by down-regulating expression of IL-6, IL-1β, iNOS and decreased free oxygen radical production (Bournival et al., 2012). Similarly, inflammation induced by oxysterols in Alzheimer disease is reduced by quercetin by down-regulating TLR4 and COX-2 signalling cascades (Testa et al., 2014). Health benefits of quercetin have been studied in age related diseases (Deepika and Paurya, 2022) and thus targeting of Sirtuin 1 (SIRT1) by quercetin has been suggested as a possible therapeutic target to treat aging-related diseases like Alzheimer, Parkinson and Huntington (Cui et al., 2022). Additionally, anti-inflammatory effect of quercetin was studied against induced atopic dermatitis (AD) in a mouse model where quercetin administration demonstrated beneficial role in controlling symptoms of AD by reducing the expression of inflammatory mediators (Hou et al., 2019, Hou et al., 2019). Further, anti-allergic function of quercetin against allergic diseases was found by inhibiting production of histamine and pro-inflammatory mediators and decreasing the release of IgE antibody by B-cells (Jafarinia et al., 2020).

Quercetin demonstrates anti-inflammatory effects by decreasing the expression of inflammatory genes such as IL-1β, COX-2, IL-6, and TNF-α in adipocytes and macrophages. This inhibition takes place by downregulating the activation of nuclear factor (NF-κB) and c-Jun N-terminal kinase. Toll-like receptors (TLRs) are critical elements of the immune system, tasked with the identification of microbial pathogens and the initiation of immune responses. The activation of TLR signaling pathways promotes the production of proinflammatory cytokines by increasing the expression of transcription factors such as NF-κB and activated protein 1 (Haddad, 2002).

Research suggests that quercetin helps modulate inflammation by influencing the TLR4/NF-κB signaling pathway. In neonatal rats with hypoxia-ischemia-induced brain injury, quercetin treatment has been found to alleviate cortical inflammation by inhibiting this pathway (Choy et al., 2019). Moreover, under standard circumstances, quercetin notably overexpresses and enhances the production of interferon-γ (IFN-γ) in T helper 1 (Th1) cells while reducing IL-4 levels in Th2 cells within peripheral blood mononuclear cells (PBMCs). Additionally, quercetin has been shown to lower the levels of inflammatory molecules, including COX-2, NF-κB, activator protein 1, mitogen-activated protein kinase (MAPK), reactive nitric oxide synthase (NOS), and C-reactive protein (CRP) (Li et al., 2016).

Quercetin and other polyphenolic compounds in certain fruits have shown promising results against the symptoms of arthritis in both experimental models and human clinical studies (Basu et al., 2018). Usage of natural compound, Quercetin has been suggested to reduce the side effects of current medication against osteoarthritis as it targets inflammatory markers (Dehkordi et al., 2023). Quercetin has also been found effective in the improvement of signs of metabolic syndrome (hyperlipidemia, obesity and hypertension) (Hosseini et al., 2021; Shabbir et al., 2021). Effective management of metabolic disorders by quercetin is mediated through multiple mechanisms (Hosseini et al., 2021). Similarly, quercetin exhibits cardioprotective effect in experimental models of cardiac injury through antioxidant and anti-inflammatory properties (Ferenczyova et al., 2020). Preclinical trials for chronic pain found analgesic role of quercetin by repressing inflammation of neurons and oxidative stress (Liu et al., 2022). Due to antiviral and anti-inflammatory properties, clinical benefits of quercetin were also studied against recent pandemic of COVID-19. Quercetin supplementation in initial stages of COVID-19 reduced the time of virus clearance, diminishing of symptoms and conversion from positive to negative test reports (Pierro et al., 2021). Though quercetin has huge therapeutic potential against diseases involving inflammation but poor oral bioavailability has been found in certain studies (Zhao et al., 2021; Grewal et al., 2021). As a result efforts have been made in the drug delivery system of quercetin to overcome problems of poor aqueous solubility and instability in physiological media to increase its applicability (Dabeek and Marra, 2019; Lee and Park, 2020).

This article will look at quercetin's therapeutic uses, with an emphasis on its anti-inflammatory qualities. It also goes over the chemistry and molecular pathways by which quercetin exerts anti-inflammatory benefits in many different kinds of inflammatory conditions. The efficacy of various nanoformulations in decreasing inflammatory conditions was also reviewed as were. It will improve the scientific community's understanding of quercetin and its anti-inflammatory properties, motivating them to develop novel therapeutic options.

## 2 Chemistry of quercetin

**Fig. 1:**
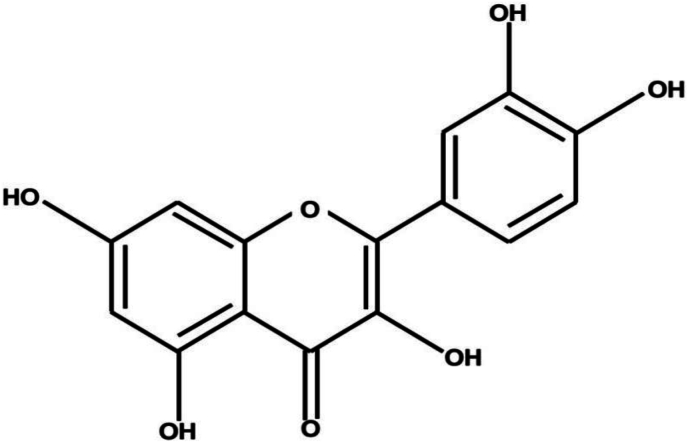
Structure of quercetin.

Quercetin (3,3′,4′,5,7-pentahydroxyflavone) is a yellow-coloured crystalline plant polyphenic flavonoid (Fig. 1), chemically it has five hydroxyl groups along with three benzene rings (Aghababaei and Hadidi, 2023; Batiha et al., 2020). It is among the most abundant dietary flavonoid present in vegetables, flowers, and fruits contributing to their characteristic colour. The bioavailability of quercetin is relatively higher compared to other phytochemicals due to its significant presence in many fruits, such as apples, red grapes, cherries, berries, and almost all citrus fruits. Apart from these, onions, broccoli, tea, red wine, olive oil, flowers, nuts, and green leafy vegetables are also great sources of quercetin (Anand David et al., 2016). Despite being widely distributed, quercetin has limited solubility in hot water and is insoluble in cold water (Azeem et al., 2023). Due to its antioxidant and anti-inflammatory effects and higher bioavailability compared to other phytochemicals, the United States Food and Drug Administration (USDA) have approved quercetin as a dietary supplement (Lu et al., 2020). Various sources of quercetin have been used for ages throughout the world, it is well known for its antiatherosclerotic, vasodilator effects, antihypercholesterolemic, antihypertensive, antiobesity, and anti-inflammatory effects (He et al., 2023). Because of its antioxidant properties, quercetin has also been shown to protect smokers' erythrocyte membranes from damage brought on by free radicals (Begum and Terao, 2002).

## 3 Absorption and metabolism of quercetin

Major absorption site for quercetin is the small intestine (Rio et al., 2013) while minor portion of it is absorbed in the stomach (Crespy et al., 2002). Within intestinal lumen, quercetin enters circulation by passing epithelial cells (Hai et al., 2020). As cellular membrane comprises of lipid bilayer so quercetin conjugates (eg. quercetin glycosides) requires the support of membrane-related transporters to pass through the membrane (Williamson et al., 2018). Multiple transporters are expressed on intestinal epithelial cells to facilitate substrate transport across gastrointestinal tract (GI) to reach circulatory system. Sodium-dependent glucose co-transporters (SGLTs) and organic anion transport polypeptides (OATPs) are the major transporters that are involved in quercetin absorption (Hai et al., 2020). SGLT-1 mediates absorption of quercetin glycosides by intestinal epithelial cells (Wolffram et al., 2002). Additionally, quercetin becomes high affinity substrate for OATP-B at lower pH whereas it is absorbed by passive diffusion at higher pH (Chabane et al., 2009). The absorption ratio of quercetin glycosides in small intestine is determined by the connected sugar moiety (Arts et al., 2004). The absorption ratio of aglycone, rutin and glucoside in which quercetin was attached with different sugar conjugate was reported to be 24 %, 17 % and 52 % respectively (Hai et al., 2020). In addition to this, deglycosylation of quercetin glycosides within intestine enhances intestinal absorption resulting in increased plasma concentration and improved bioavailability (Arts et al., 2004). This process of deglycosylation is mediated by lactase-phlorizin hydrolase (LPH) (Hai et al., 2020). Quercetin glycosides are hydrolyzed by LPH to release quercetin aglycone which is mainly absorbed by passive diffusion (Nemeth et al., 2003). These quercetin glycosides can also be hydrolyzed by cytosolic β-glucosidase (CBG) after SGLT-1 mediated absorption by epithelial cells (Wolffram et al., 2002). Rutin is hardly absorbed by the small intestine as it is not a substrate for LPH or CBG. As a result its absorption occurs at distal part of GI after degradation by intestinal microbes (Cermak et al., 2006; Russo et al., 2012). This is the main reason behind lower absorption rate of rutin compared to other glycosides (Hai et al., 2020). The gut microbiota including strains of *Streptococcus*, *Lactobacillus*, *Bifidobacterium* and *Bacteroides* produces α-rhamnosidases and β-glucosidases that performs deglycosylation of rutin to form aglycone which is later on passed into circulation for further catabolic reactions to form lower molecular weight compounds (Cermak et al., 2006; Almeida et al., 2018). The bioaccumulation and bioavailability of quercetin can further be affected by quercetin prenylation and high dietary fat consumption (Terao, 2017).

Absorption of quercetin is followed by phase II metabolism within small intestine (Boersma et al., 2002) which involves reactions mediated by Sulfotransferases (SULTs), Uridine-5′-diphosphate Glucuronosyl Transferases (UGTs) and Catechol-O-Methyl Transferases (COMTs) (Almeida et al., 2018; Hai et al., 2020). This results in glucuronidated and sulphated/methylated metaboliting conjugations. The UGT-mediated glucuronidation of quercetin within liver and intestine is considered as one of the most significant quercetin metabolic pathway (Zhang et al., 2007). Quercetin glucuronidation finds the involvement of UGT1A9 in human liver while similar process is mediated by UGT1A1 and UGT 1A8 in human intestine (Boersma et al., 2002). Quercetin phase II metabolites have increased hydrophilicity resulting in reduced membrane transport. As a result specific transporters are involved to deliver these metabolites to gut lumen or bloodstream (Petri et al., 2003). Efflux of these substrates is performed by ATP-binding cassette (ABC) transporters (Dreisietel et al., 2009; Haribar and Ulrih, 2014). Anionic metabolites are effluxed by multidrug resistance proteins (MDRs) belonging to ABC superfamily (Hoffmann and Kroemer, 2004). MRP2 transporter reduces the bioavailability of absorbed quercetin by transporting it back into gut lumen (Chabane et al., 2009; Hai et al., 2020). Additionally, breast cancer resistance protein (BCRP) effectively caused efflux of quercetin metabolite in *in-situ* system (Sesink et al., 2005; Song et al., 2020). The transport of quercetin metabolites to the serosal side is regulated by MRPs (Zhang et al., 2007).

Pathways for quercetin metabolism are dependent upon conjugating enzymes which have known genetic polymorphisms and can be induced by drugs, food and the environment. In addition to this, catabolism of quercetin is affected by microbiota composition which itself is influenced by multiple factors. These factors can cause substantial inter-individual variation in quercetin absorption and metabolism (Almeida et al., 2018). The same has been observed for polyphenols where metabolism of ellagitannins in humans showed several metabotypes (Sarrias et al., 2017). Considering such variations, pharmacogenomics studies have categorized individuals into poor, intermediate and extensive metabolizers. As a result, similar sort of inter-individual variation can be observed in the bioavailability of quercetin (Almeida et al., 2018).

## 4 Mechanistic insights

An inflammatory biological reaction occurs when a human body is subjected to damaging or irritating stimuli. This response aids in self-defence by attempting to eliminate pathogens, damaged cells, or other harmful stimuli while also initiating healing. It's not always the case that inflammation means infection. Most of the time, a fungus, bacteria, or virus causes a disease, whereas the body's attempt to cure itself is what causes inflammation. Macrophages are the main cells responsible for chronic inflammation during inflammatory diseases. They overproduce pro-inflammatory cytokines, prostaglandin E2 (PGE2), and nitric oxide (NO) which are critical to the outcomes of inflammation. Mitogen-activated protein kinase (MAPK) and nuclear factor-kappa B pathways have been proposed as important mechanisms for the regulation of inflammatory mediator expressions, despite the complexity of the cellular signaling networks controlling inflammation (Hisanaga et al., 2016; Haddad, 2002). When bacterial *lipopolysaccharide* (LPS) activates macrophages, MAPK can increase the release of inflammatory mediators such as cyclooxygenase-2 (COX-2) and inducible nitric oxide synthase (iNOS), as well as cytokines. Furthermore, recent studies have demonstrated that polyphenolic substances have the ability to directly bind to MAPK proteins in order to decrease kinase signalling (Rossol et al., 2011; Chang and Karin, 2001). The modulation of inflammation is one of quercetin's most notable core capabilities. Quercetin reduces pro-inflammatory mediators such as prostaglandins and leukotrienes by inhibiting the inflammatory enzymes lipooxygenase and cyclooxygenase (COX) (Anand David et al., 2016; Xiao et al., 2011).

High levels of C reactive protein (CRP) have been linked to a number of disease conditions, including heart disease, and obesity. In human hepatocyte-derived cell lines, quercetin significantly lowered the levels of inflammatory mediators such as nitric oxide (NO) synthase, cyclooxygenase-2 (COX 2), and CRP. Rats treated with 80 mg of quercetin showed a considerable antiarthritic effect against adjuvant-induced arthritis, as well as inhibition of both acute and chronic inflammation (Guardia et al., 2001; García-Mediavilla et al., 2007; Mamani-Matsuda et al., 2006). Numerous *in vitro* studies have shown that quercetin suppresses the proliferation of interleukin (IL)-8-induced lipopolysaccharide (LPS) in lung A549 cells as well as the production of LPS-mediated tumor necrosis factor (TNF-α) in macrophages. Furthermore, quercetin can reduce the levels of IL-1α and TNF-α generated by LPS, which reduces the amount of apoptotic neuronal cell death triggered by activated microglia (Aghababaei and Hadidi, 2023; Li et al., 2016; Bureau et al., 2008).

A few studies reported that quercetin also decreased the immunoexpression of IL-17 and TNF-α in mice with arthritis (Costa et al., 2021). Through the control of immune cells, inflammatory mediators including IL-1β, IL-6, IL-17, and TNF-α, and matrix metalloproteinases, quercetin lowers inflammation in rheumatoid arthritis. In addition, a small number of studies found that this flavonoid raises IL-10 and other pro-inflammatory cytokine levels. Patients with rheumatoid arthritis are linked to elevated liver transaminase levels and changes in the liver morphology. Rheumatoid arthritis-related hepatotoxicity is decreased by quercetin treatment, indicating a potential hepatoprotective benefit (Kawaguchi et al., 2019).

**Fig. 2:**
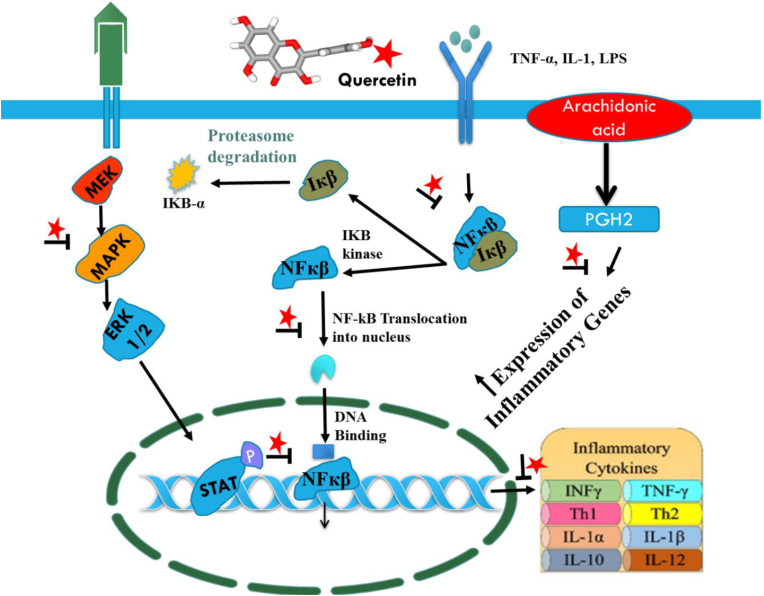
Representation of the mechanism insights of quercetin against several inflammatory conditions.

In rats with acute pancreatitis caused by hypertriglyceridemia, quercetin demonstrated an anti-inflammatory response by lowering TNF-α, IL-1β, NF-κB, and IL-6, which therefore decreased histopathological damage (Choy et al., 2019; Al-Khayri et al., 2022). Additionally, it has been demonstrated to improve the 5′ adenosine monophosphate-activated protein kinase (AMPK) pathway, which in turn promotes the production of glucose transporter type 4 (GLUT4). Experiments conducted on animals have demonstrated a decrease in blood glucose levels in response to quercetin treatments at doses of 10, 25, and 50 mg/kg body weight. Additionally, it was shown to lower GLUT2 absorption of glucose, lipid peroxidation, and insulin-dependent PI3K activation (Bule et al., 2019; Al-Ishaq et al., 2019). A number of cell line studies in corroboration with other animal studies have validated that quercetin can modulate the activity of multiple mechanistic targets to treat acute and chronic inflammatory situations and have been explained in Fig. 2 and Table 1.

**Table 1:** Anti-inflammatory studies of quercetin in various diseases.

Associated Disease	Study Modal (both In vitro and in Vivo)	Mechanisms/Molecular Targets	Concentration	References
Global cerebral ischemia	Sprague‒Dawley (SD) rats	↓ neurological impairment, ↑ learning and memory abilities, ↓‘anxiety, ↓ neuronal injury ↓ brain edema, ↓ microglial activation, ↓ TLR4 ↓TRIF, ↓ IL-1β, ↓ TNF-α	10, 30, 50 mg/kg	Wang et al. (2024)
Global cerebral ischemia	BV2 cells	↓TNF-α,↓IL-1β↑ IL-4, ↑IL-10	0, 10, 20, 30, 40 μM	Wang et al. (2024)
Lung inflammation	Macrophages	↓ p-PI3K, ↓p-AKT, ↓ p-IκBα, ↓p-NF-κB p65, ↓neutrophil infiltration, ↓IL-1β, ↓IL-6, ↓TNF- α	–	Jia et al. (2024)
Respiratory syncytial virus (RSV) infection disease	BALB/c mice	↓ glycolysis and TCA metabolism, ↓ SDH, ↓Hif-1α/NLRP3 signaling,	0, 30, 60, 120 mg/kg	An et al. (2024)
Avian chronic respiratory disease	Chickens	↓IL-1β, ↓ IL-6,↓TNF-α, ↑ respiratory inflammation injury, ↑p- AMPK, ↑ SIRT1 ↓p-P65	50 mg/kg	Lu et al. (2023)
Depression	Mice	↓ immobility time, ↓ swimming and climbing time (forced swim test), ↑ head dips, ↑ time spent, and ↑ entries in open arm elevated plus maze test, ↓ ALP and ALT, ↓Caspase-3, ↑ anti-inflammatory and anti-oxidation(hippocampus and prefrontal cortex)	0,40, 80 mg/kg	Ge et al., 2023
Keratitis	RAW264.7 cells	↓ *A. fumigatus* growth and adhesion, ↓ macrophage infiltration in the mouse cornea, ↓TLR-4, ↓IL-1β, ↓TNF-α, ↓ IL-6	32 μM	Luan et al. (2023)
Pyroptosis	THP-1 cells	↓ NLRP3, ↓cleaved-caspase1, ↓ IL-1β, ↓N-GSDMD, ↓ ROS, ↓p- P65, ↓translocation from cytoplasm into nuclear, ↓ TLR2/Myd88, ↓p-AMPK	–	Luo et al., 2022
Gastric injury	GES-1 cells	↓TNF-α, ↓p- c-Src, ↓p-ERK1/2, ↓ p-c-Fos, ↓p- p65, ↓ MMP-9	0, 0.01, 0.1, 1, or 10 mM	Hsieh et al. (2022)
Lung injury	C57BL/6J mice	↓ lung inflammation, ↓ alveolar wall destruction, ↓ lactate synthesis,↑SIRT1, ↓ NLRP3 inflammasome, ↓ TNFα, ↓IL-1β, ↓ IL-6	50 mg/kg	Chen et al. (2022)
Anti-inflammatory assay	RAW 264.7 cells	↓TNF-α, ↓IL-6, ↓ROS, ↓nitric oxide,	10 μM	Shanmugasundaram and Roza (2022)
Chronic DSS-induced colitis	C57BL/6 mice	↓ Severe outcome and clinical symptoms of DSS Colitis, ↓ DSS-induced inflammation, ↓ bloody lesions, ↓ abscesses, ↓permeability of the intestinal tissue, ↓ tissue inflammation, ↓ MPO expression, restored Claudin-1 expression	50 mg/kg	Riemschneider et al. (2021)
Liver injury	SD rats	↓P53, ↓Bax, ↓ cleaved-cas3, ↓ Bcl-2,↓ ALT/AST, ↓apoptosis, ↓ NLRP3	0, 50,100 mg/kg	Zhang et al. (2020)
Retinal inflammation	Retinal pigment epithelial cells (ARPE-19 cells)	↓ p-PKCδ, ↓p-JNK1/2, ↓p- ERK1/2, ↓ ICAM-1, ↓ MMP-9	0, 5, 10, 50 μM	Cheng et al. (2019)
Atopic dermatitis	C57BL/6 mice	↓CCL17, ↓CCL22, ↓ IL-4, ↓ IL-6, ↓IFN-γ, ↓TNF-α, ↓ AD skin lesions	1 % (Quercetin) topical cream	Hou et al. (2019)
Atopic dermatitis	Human keratinocyte HaCaT cells	↓CCL17, ↓CCL22, ↓IFN-γ, ↓TNF-α, ↓ MDC, ↓TARC	0, 30, 90 μM	Hou et al. (2019)
Spinal cord injury	Sprague-Dawley rats	↑functional recovery, ↓ necroptosis, ↓ myelin and axonal loss, ↓TNFα,↓ iNOS, ↓ CD86, ↓ TNF-α, ↓IL-12, ↓IL-1β, ↓ iNOS, ↓pSTAT1, ↓NF- κB, ↓p-NF-κB	–	Fan et al. (2019)
Anti-inflammatory assay	Raw 264.7 cells	↓TNF-α, ↓IL-1β, ↓ IL-6	15, 22.4 μg/mL	Tang et al., 2019
Anti-inflammatory assay	RAW 264.7 cells	↓NO, ↓PGE2, ↓iNOS, ↓COX-2, ↓ TNF-α, ↓IL-12, ↓IL-1β, ↓ TNF-α, ↓IL-12, ↓IL-1β IL-6, ↓ TNF-α, ↓IL-12, ↓IL-1β GM-CSF	0,2.5,5.0,10.0 μM	Endale et al. (2013)

## 5 Nanoformulations of quercetin to inhibit inflammation

**Fig. 3:**
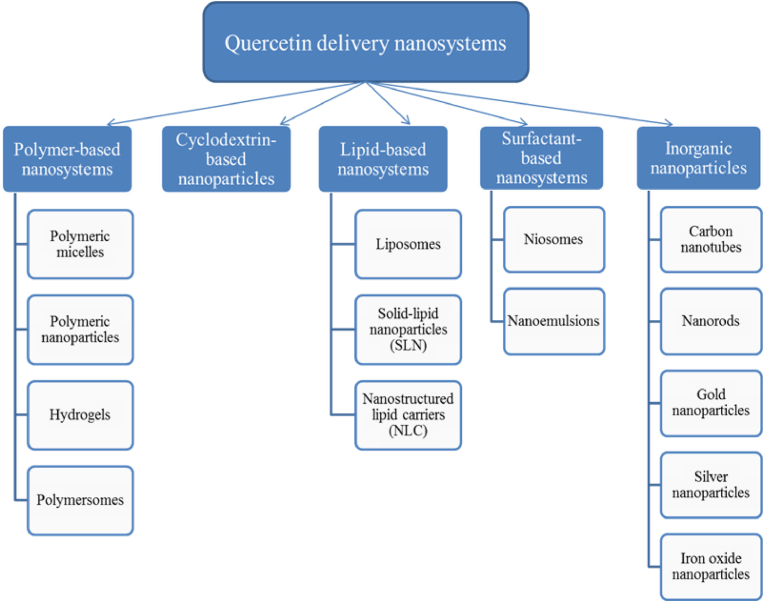
Illustration of various quercetin delivery nanosystems.

Quercetin has been widely tested against diseases like tumor, diabetes, obesity, neurological and cardiovascular diseases because of its vast therapeutic potential including antioxidant and anti-inflammatory properties (Ulusoy and Sanlier, 2020; Zang et al., 2021). But poor solubility and lower bioavailability of quercetin has limited its clinical application (Moradi et al., 2020; Tomou et al., 2023, Tomou et al., 2023; Zhang et al., 2023). As a result different nanosystems (Fig. 3) were targeted to improve the bioavailability and efficacy of quercetin (Riva et al., 2019; Ebrahimpour et al., 2020; Tomou et al., 2023). Most of these systems are biocompatible and appropriate for delivery with controlled release enhancing the Absorption, Distribution, Metabolism, Excretion and Toxicology (ADME(T)) profile of encapsulated active pharmaceutical ingredients (APIs). Each of these nanoformulations has its own advantages and limitations for drug delivery (Tomou et al., 2023).

Different formulations of quercetin have been studied to target wound healing, metabolic syndrome, neurodegenerative disorders, tumor and respiratory viral infections (Hatahet et al., 2016; Taghipour et al., 2019; Moradi et al., 2020; Zang et al., 2021; Mariano et al., 2023) (Table 1). Skin is continuously exposed to oxidising agents and inflammogens due to environmental exposure. Quercetin can support skin regeneration in wound healing because of its antioxidant and anti-inflammatory potential. It strongly inhibits action of NF-kB and release of pro-inflammatory cytokines making it potential candidate to target chronic wounds. Additionally, quercetin possesses anti-aging action on keratinocytes and whitening effect on skin. But because of poor solubility, skin penetration ability of quercetin is limited. As a result, different formulation approaches were targeted to increase its dermal penetration (Hatahet et al., 2016). Quercetin formulation in nanodosage forms was prepared to overcome its topical limit penetration ability and increase its stability. Various targeted nanodosage formulations of quercetin included nanoemulsions (Fasolo et al., 2009), liposomes (Chessa et al., 2011), lipid nanoparticles (Scalia et al., 2013), Nanostructured Lipid Carriers (NLC), Solid Lipid Nanoparticles (SLN) (Bose et al., 2013; Bose and Kohn, 2013) and mesoporous silica (Sapino et al., 2015). No transdermal delivery of quercetin was observed with novel dosage forms due to poor water solubility, selective lipophilicity and stratum corneum barrier severely affecting the penetration depth of quercetin in skin layers. Further research can increase the application of quercetin to target skin disorders like psoriasis and atopic dermatitis (Hatahet et al., 2016). Neurodegenerative and other related disorders are also targeted using nanoformulations developed from variety of natural products. Main obstacle in the path of treatment strategies for neurodegenerative disorders is the presence of blood brain barrier (BBB). Out of the different nano-methods that have been targeted to dissipate this problem, polymeric nanoparticles (PNPs) performed as one of the best drug delivery carriers. PNPs generated great interest because of high drug loading capacity, longer half-life in circulation and greater drug protection capacity against debasement (Moradi et al., 2020). Current studies on nanoparticles based drug delivery systems have claimed that nanomaterials can pass the BBB either through non-invasive or invasive mechanism. Invasive mechanism involves rupturing of BBB and transportation of nanomaterials across BBB through paracellular pathways like intracerebral injection (Moradi et al., 2020). Non-invasive strategy preserves the basic structure of BBB (Xie et al., 2019). One of the non-invasive approaches involves encapsulation of the drug inside nanocarriers for simplified entry of drug into brain (Pooviah et al., 2018). Studies on therapeutic potential of 2-hydroxypropyl-β-cyclodextrin (HP-β-CD) against Niemann-Pick disease type C (NPC1) characterized by severe neuronal injury in mice model identified that intracerebroventricular administration of HP-β-CD inhibited cerebellar Purkinje cell damage with significant reduction in biomarker levels (Fukaura et al., 2021). Another study on HP-β-CD to target NPC1 in mice model highlighted the fact that HP-β-CD cannot cross BBB in significant amounts. As a result it has to be administered at very high doses either subcutaneously or intraperitoneally that itself raised concerns regarding pulmonary toxicity (Calias, 2017). Targeting of various neurodegenerative diseases through Quercetin based nanoformulations is underway but studies on Qu-loaded nanoformulations is mostly restricted to pre-clinical level due to poor loading and stability of drug formulation, along with poor scale-up capacity (Vishwas et al., 2023). Varieties of nanosystems for delivering therapeutic chemicals including quercetin against Alzheimer's disease (AD) are targeted for delivery across BBB to exert neuroprotective effect (Naser et al., 2023; Pei et al., 2025). Similarly, effect of quercetin against another neurodegenerative disease either in free or nanosystems formulation through varied routes of administration showed potential usage against Parkinson's disease by inhibiting oxidative stress and neuroinflammatory response (Vian et al., 2024).

Additionally, Different nanoformulations of quercetin (QC) like nanocapsules, liposomes and microsphere have been suggested (Attar et al., 2023; Moradi et al., 2020). Out of these, QC-nanocapsulation was found to be most potent (Ghaffari et al., 2018) and quercetin phytosomes attracted greater interest (Attar et al., 2023). Nanoformulations of quercetin with carrier molecules (liposomes, polymeric micelles, PLGA nanoparticles, silica nanoparticles, carbon based nanoparticles etc.) have also been studied for tumour therapy by enhancing drug accumulation through P-gp down-regulation, mitochondrial dysfunction, autophagy, apoptosis and cell cycle arrest in addition to function related to antioxidant capability (Zang et al., 2021). Similarly, different nanoformulations of phytochemicals were studied for management of metabolic syndrome that can increase the risk of other diseases (Taghipour et al., 2019). Administration of quercetin can target NO synthase activation to decrease blood pressure and cholesterol level (Rivera et al., 2008). Some of the nano-formulation studied to treat chronic inflammation in various diseases is discussed in Table 2.

**Table 2:** Quercetin-based nanoformulations to target metabolic syndrome.

Nano-formulation	Disorder/Disease	Cell/Animal model	Dosage	Size	Effect	Reference
PGLA NPs (QU-NP)	Diabetes	Streptozotocin (STZ)-induced diabetic rats	150 mg/kg	179.9 ± 11.2 nm	Increased levels of CAT and SOD, Decreased dose of drug	Chitkara et al., 2012
Nanoemulsion	Oxaliplatin-induced toxicity	BALB/c mice	20 mg/kg	_	Decreased inflammation, Prevented induced neuro-and hepato-toxicity	Schwingel et al. (2014)
Quercetin nanorods	Diabetes	Alloxan-induced diabetic rats	20 mg/kg	15.4 nm	Decreased G6Pase, SOD, CAT, AST, ALP, ALT	Alam et al., 2016
(QUE/P) NP	Diabetic nephropathy	Diabetic rats	10 mg/kg	32 nm	Decreased expression of ICAM-1	Tong et al., 2017
Chitosan-alginate core-shell (pH sensitive)	Diabetes	Human colonic epithelial cell line (HT29) and Streptozotocin (STZ)-induced diabetic rats	100 mg/kg	91.58 nm	Decreased levels of AST, ALT and ALP in serum	Mukhopadhyay et al. (2018)
Quercetin conjugated-iron oxide NPs	H_2_O_2_ induced cytotoxicity	PC12 cells	100–1000 μg/ml	72.9 nm	Antioxidant, anti-inflammatory and anti-apoptotic effects	Yarjanli et al. (2019)
Quercetin conjugated superparamagnetic iron oxide nanoparticles	Diabetes-induced memory impairment	Diabetic rats	25 mg/kg	30–50 nm	Down-regulated NF-kB pathway, reversed neuroinflammation and memory impairment	Ebrahimpour et al. (2020)
Quercetin-entrapped liposomes	Doxorubicin-induced toxicity	Human umbilical vein endothelial cells (HUVECs)	0.001–100 μg/ml	20 nm	Inhibited oxidative stress and inflammation, reduced apoptosis and increased cell viability	Muresan et al. (2021)
Quercetin niosomal system	Carrageenan-induced paw edema	Rat model	10 ml/kg	231.07 ± 8.39 nm	Anti-inflammatory action	Ghadi et al., 2021
Quercetin -incorporated micelles	Tumor	MCF-7 cell line	_	22 nm	Increased oral bioavailability, antioxidant activity and cell viability	Patel et al., 2022
Quercetin in silver nanoparticles	Cutaneous Leishmaniasis lesions	L. major infected BALB/c mice	_	113.9 nm	Decreased inflammatory response, increased wound healing	Alemzadeh et al. (2022)
Quercetin polymeric nanoparticles	Acute Kidney injury (AKI)	I/R induced AKI mouse model	_	21 nm	Decreased inflammation, oxidative stress and renal degradation	Huang et al. (2022)
Quercetin liposomes	Hepatic Ischemia and reperfusion injury (IRI)	Rat model of hepatic IRI	1.3 mg/kg	0.12 ± 0.01 μm	Decreased inflammatory markers and enhanced recovery	Silva et al., 2022
Quercetin liposomes	Allergy	RBL-2H3 cells	_	_	Decreased release of histamine and reduced expression of inflammatory factors	Zhang et al., 2023

## 6 Quercetin as lipid-based nanosystem

**Fig. 4:**
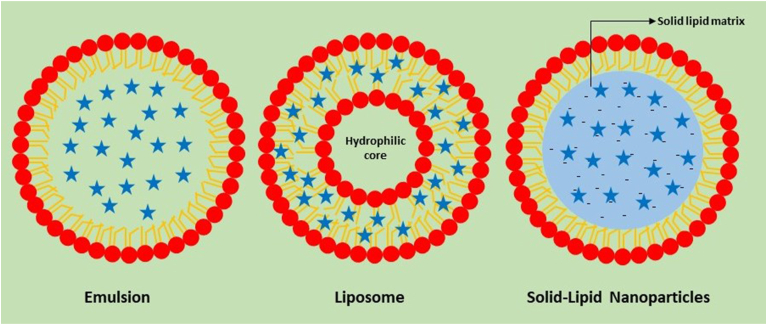
General view of lipid based nanoparticles https://www.pharmaexcipients.com/news/encapsulating-cannabinoids-lnp-part-2/

This system includes the use of lipososmes, SLN and NLC (Fig. 4). Nano lipidic carriers (NLCs) loaded with QC enhanced bioavailability, antioxidant activity and delivery to brain (Kumar et al., 2016). Improvement in neuronal damage induced by ischemia reperfusion was observed in *vivo* studies targeting nanoencapsulated QC by increasing neuronal count and elevating antioxidant activity (Ghosh et al., 2013). *In vitro* studies highlighted that QC-SLNs ameliorated neurotoxicity whereas it improved memory in studied animal models of dementia and Alzheimer disease (AD) (Dhawan et al., 2011). Another liposomal structure loaded with QC crossed blood brain barrier (BBB) and recovered neurotoxicity in AD model (Kuo et al., 2018). Administration of QC liposomes through nasal route decreased cholinergic neurons degeneration in animal model for AD by decreasing oxidative stress (Moradi et al., 2020). Similar study on quercetin liposomal formulation (SPC_Querc) showed effective results at both *in vitro* and *in vivo* level against Ischemia and reperfusion injury (IRI) by decreasing inflammation markers and enhancing recovery (Silva et al., 2022). Application of quercetin against respiratory viral infections is effective because of its anti-inflammatory mechanism by inhibiting metabolism and release of mediators (Mariano et al., 2023). These functions proved significant to counter hyper-inflammation of the lungs caused by viral infections. Encapsulation of quercetin in liposomes improved solubility and increased bioavailability in lungs thereby reducing the administration dose and possible side effects in murine models (Yuan et al., 2006). Additionally, quercetin liposome reduced concentration of inflammatory cells, plasma TNF-α and TGF-β1 and increased antioxidant levels within lungs of murine model (Liu et al., 2013).

## 7 Quercetin as polymer-based nanosystem

**Fig. 5:**
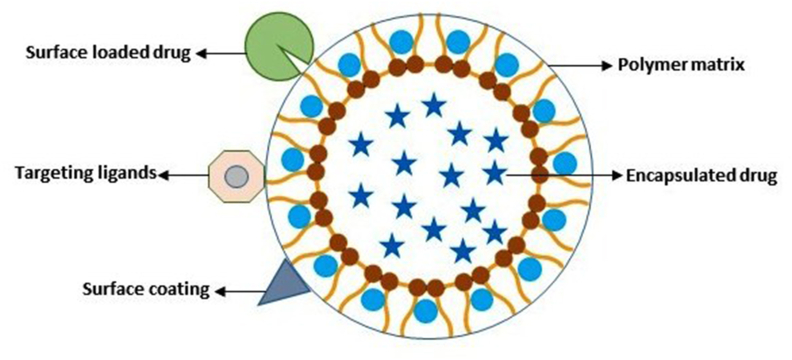
General view of polymer based nanoparticles.

QC loaded in polymeric nanocapsules enhanced brain uptake, bioavailability and mitochondrial localization (Ghosh et al., 2017). Loading of quercetin on PLGA increased its bioavailability and decreased required dose to target diabetes in rats when administered orally (Chitkara et al., 2012). Quercetin-encapsulated polymeric NPs (Fig. 5) were studied as an effective approach for COVID-19 treatment as it was found to be effective on mucin protein responsible for removal of virus/airborne particles from the lung (DeMarino et al., 2017; Neufurth et al., 2021). QC nanoformulation comprising of chitosan-alginate proved non-toxic and thus can be used as a biocompatible carrier for oral administration (Mukhopadhyay et al., 2018). Hyaluronic acid-quercetin-conjugated silver nanoparticles increased anti-cancerous efficacy of quercetin by delivering the drug to precise tumor location (Al-Serwi et al., 2023). Polyamidoamine (PAMAM) dendrimers as drug delivery carriers for quercetin showed sustained release, more stability and improved anti-inflammatory activity in rats. As a result, PAMAM-based dendrimers can be targeted as suitable polymeric nanocarrier for vast applications (Maddan et al., 2016). Nanomedicine formulation of bioactive phosphorous dendrimer based co-delivery system loaded with both catalase and quercetin showed promising results against osteoarthritis through cooperative macrophage reprogramming (Sun et al., 2025).

## 8 Quercetin as additional nano systems

Quercetin nanoemulsion decreased inflammation, pain, apoptosis and checked oxaliplatin induced toxicity in mice (Schwingel et al., 2014). Quercetin nanorods (15.4 nm) tested on alloxan-induced diabetic rats decreased fasting blood glucose level. It further enhanced concentration of antioxidant enzymes, diminished protein oxidation and decreased level of functional markers of both kidney and liver in diabetic mice (Alam et al., 2016). Phytosome nanoparticles loaded with quercetin were found as an encouraging hormone replacement therapy (El-Fattah et al., 2017).

## 9 Synergistic effects of quercetin in combination therapies for cancer

Studies have highlighted the possible addition of quercetin to conventional therapies and synergistic effects with other nutraceuticals or targeted treatments. Quercetin has been shown to increase the anti-cancer effects of curcumin, resveratrol, and green tea polyphenols, enhancing apoptosis and diminuting tumor development in different models (Joshi et al., 2023). Furthermore, it has been found to enhance the sensitivity of cancer cells to chemotherapeutic agents like doxorubicin and cisplatin, potentially leading to lower dosages and reduced side effects without compromising efficacy (Xu et al., 2021).

Furthermore, research indicates that quercetin increases the sensitivity of cancer cells to chemotherapy and radiotherapy, possibly resulting in better treatment outcomes. It has also been shown to enhance the efficacy of some targeted approaches including tyrosine kinase inhibitors adding to their anti-cancer effects (Ge et al., 2023, Ge et al., 2023). Numerous preclinical studies showed that quercetin augment the effectiveness of routine chemotherapeutics as it enhances the sensitivity of the cancerous cells as well as the toxicity. Additionally, the bioactive nature of it may prevent some of the detrimental effects tied to common cancer treatments which will help positively impact a patient's overall health and quality of life.

## 10 Limitations and safety concerns associated with quercetin nano-formulations

The polyphenolic flavonoid quercetin has received significant attention for its possible therapeutic applications, ranging from anti-inflammatory, anti-cancer to antioxidant properties. Quercetin nanoformulations have emerged as a possible solution to these limitations, leading to its improved bioavailability and therapeutic efficacy. However, poor bioavailability, limited solubility, and rapid metabolism hinder its use in clinical settings (Katiúscia et al., 2022). These nanoformulations (including liposomes, nanoparticles, and micelles) exhibit boosted pharmacokinetics, enhanced cellular uptake, and targeted delivery to pathological tissues. Despite the promising preclinical results, many translational challenges need to be overcome to realize the clinical promise of quercetin and its nanoformulations (Chen et al., 2022; Patel et al., 2022).The challenges encompass scalability, stability, regulatory obstacles, the necessity for standardized manufacturing processes, and stringent safety assessments. Furthermore, the ideal design of nanoformulations, dosing schedules, and biomarkers for therapeutic monitoring necessitates additional research.

The increasing use of quercetin and its nanoformulations raised questions about their safety (Tomou et al., 2023). High doses of quercetin have been shown in numerous studies to cause gastrointestinal disturbances, interact with certain medications, and exacerbate renal and hepatic injury (Tomou et al., 2023; Dechsupa et al., 2022). In addition, even nanoformulations of quercetin developed to enhance bioavailability and bioactivity may increase toxicity by virtue of their enhanced systemic exposure. Quercetin in nanoparticulate form induced oxidative stress, inflammation and cytotoxicity in various immune and non-immune cell lines and animal models (Katiúscia et al., 2022). Moreover, the lack of uniformity in the nests' preparation and characterisation of quercetin nanoformulations is a chief concern raising issues of batch-to-batch variation and contamination (Dong et al., 2021). This review underscores the necessity for additional research on the safety and toxicity of quercetin and its nanoformulations, stressing the significance of thorough testing and regulation to guarantee their safe application in human populations.

Consequently, there is a necessity to investigate the clinical applications and translational obstacles of quercetin and its nanoformulations, emphasizing their potential in oncology, cardiovascular conditions, and neurodegenerative disorders. Addressing the aforementioned challenges and limitations will facilitate the successful translation of quercetin-based nanoformulations into clinical practice, ultimately enhancing patient outcomes and quality of life.

## 11 Conclusion and future prospective

This review study represents the information available regarding the anti-inflammatory properties of quercetin nano-formulations in various preclinical animals. Research indicates that quercetin's significant anti-inflammatory properties have been linked to several medical uses. This provides more support for the use of quercetin in the investigation of new lead compounds. It is anticipated that creating quercetin's nanoformulations would help overcome its bottlenecks and soon result in the creation of safer and more effective anti-inflammatory medications. Investigating the anti-inflammatory mechanisms of formulations based on quercetin is imperative to combat the global trend of infection cases rising steadily. Computational drug discoveries could also play an important role in revealing hidden cellular interactions of quercetin in the inflammatory microenvironment of the tissue. Additionally, combining these novel compounds with traditional synthetic medications may improve therapy responses and lower their effective concentrations, enhancing the quality of life for patients with various cancers.

## CRediT authorship contribution statement

**Diwakar Aggarwal:** Writing – original draft. **Mayank Chaudhary:** Writing – original draft. **Sachin Kumar Mandotra:** Writing – original draft. **Hardeep Singh Tuli:** Writing – review & editing, Conceptualization. **Ritu Chauhan:** Writing – original draft. **Naveen Chandra Joshi:** Writing – review & editing, Conceptualization. **Damandeep Kaur:** Writing – review & editing, Conceptualization. **Laurent Dufossé:** Writing – review & editing, Conceptualization. **Abhishek Chauhan:** Writing – review & editing, Conceptualization.

## Declaration of competing interest

The authors declare that they have no known competing financial interests or personal relationships that could have appeared to influence the work reported in this paper.

## Data Availability

No data was used for the research described in the article.

## References

[bib1] Aghababaei F., Hadidi M. (2023). Recent advances in potential health benefits of quercetin. Pharmaceuticals.

[bib2] Al-Ishaq R.K., Abotaleb M., Kubatka P., Kajo K., Büsselberg D. (2019). Flavonoids and their anti-diabetic effects: cellular mechanisms and effects to improve blood sugar levels. Biomolecules.

[bib3] Al-Khayri J.M., Sahana G.R., Nagella P., Joseph B.V., Alessa F.M., Al-Mssallem M.Q. (2022). Flavonoids as potential anti-inflammatory molecules: a review. Molecules.

[bib4] Al-Serwi R.H., Eladl M.A., El-Sherbiny M., Saleh M.A., Othman G., Alshahrani S.M., Alnefaie R., Jan A.M., Alnasser S.M., Albalawi A.E., Mohamed J.M.M., Menaa F. (2023). Targeted drug administration onto cancer cells using hyaluronic acid-quercetin-conjugated silver nanoparticles. Molecules.

[bib5] Alam M.M., Abdullah K.M., Singh B.R., Naqvi A.H., Nassem I. (2016). Ameliorative effect of quercetin nanorods on diabetic mice: mechanistic and therapeutic strategies. RSC Adv..

[bib6] Alemzadeh E., Karamian M., Abedi F., Bojd M.Y.H. (2022). Topical treatment of cutaneous leishmaniasis lesions using quercetin/Artemisia-capped silver nanoparticles ointment: modulation of inflammatory response. Acta Trop..

[bib7] Almeida A.F., Borge G.I.A., Piskula M., Tudose A., Tudoreanu L., Valentova K., Williamson G., Santos C.N. (2018). Bioavailability of quercetin in humans with a focus on interindividual variation. Compr. Rev. Food Sci. Food Saf..

[bib8] An L., Zhai Q., Tao K., Xiong Y., Ou W., Yu Z., Yang X., Ji J., Lu M. (2024). Quercetin induces itaconic acid-mediated M1/M2 alveolar macrophages polarization in respiratory syncytial virus infection. Phytomedicine.

[bib9] Anand David A.V., Arulmoli R., Parasuraman S. (2016). Overviews of biological importance of quercetin: a bioactive flavonoid. Pharmacogn. Rev..

[bib10] Arts I.C.W., Sesink A.L.A., Peters M.F., Hollman P.C.H. (2004). The type of sugar moiety is a major determinant of the small intestinal uptake and subsequent biliary excretion of dietary quercetin glycosides. Br. J. Nutr..

[bib11] Attar E.S., Chaudhari V.H., Deokar C.G., Dyawanapelly S., Devarajan P.V. (2023). Nano drug delivery strategies for an oral bioenhanced quercetin formulation. Eur. J. Drug Metab. Pharmacokinet..

[bib12] Azeem M., Hanif M., Mahmood K., Ameer N., Chughtai F.R.S., Abid U. (2023). An insight into anticancer, antioxidant, antimicrobial, antidiabetic and anti-inflammatory effects of quercetin: a review. Polym. Bull..

[bib13] Basu A., Schell J., Scofield R.H. (2018). Dietary fruits and arthritis. Food Funct..

[bib14] Batiha G.E.S., Beshbishy A.M., Ikram M., Mulla Z.S., El-Hack M.E.A., Taha A.E., Elewa Y.H.A. (2020). The pharmacological activity, biochemical properties, and pharmacokinetics of the major natural polyphenolic flavonoid: quercetin. Foods.

[bib15] Begum A.N., Terao J. (2002). Protective effect of quercetin against cigarette tar extract-induced impairment of erythrocyte deformability. J. Nutr. Biochem..

[bib16] Bhaskar S., Sudhakaran P.R., Helen A. (2016). Quercetin attenuates atherosclerotic inflammation and adhesion molecule expression by modulating TLR-NF-kB signaling pathway. Cell. Immunol..

[bib17] Boersma M.G., van der Woude H., Bogaards J., Boeren S., Vervoort J., Cnubben N.H.P., van Iersel M.L.P.S., van Bladeren P.J., Rietjens I.M.C.M. (2002). Regioselectivity of phase II metabolism of luteolin and quercetin by UDP-glucuronosyl transferases. Chem. Res. Toxicol..

[bib18] Bose S., Kohn B.M. (2013). Preparation and characterization of lipid based nanosystems for topical delivery of quercetin. Eur. J. Pharmaceut. Sci..

[bib19] Bose S., Du Y., Takhistov P., Kohn B.M. (2013). Formulation optimization and topical delivery of quercetin from solid lipid based nanosystems. Int. J. Pharm..

[bib20] Bournival J., Plouffe M., Renaud J., Provencher C., Martinoli M.G. (2012). Quercetin and sesamin protect dopaminergic cells from MPP+ -induced neuroinflammation in a microglial (N9)-neuronal (P12) coculture system, Qxid. Med. Cell Longev..

[bib21] Bule M., Abdurahman A., Nikfar S., Abdollahi M., Amini M. (2019). Antidiabetic effect of quercetin: a systematic review and meta-analysis of animal studies. Food Chem. Toxicol..

[bib22] Bureau G., Longpré F., Martinoli M.G. (2008). Resveratrol and quercetin, two natural polyphenols, reduce apoptotic neuronal cell death induced by neuroinflammation. J. Neurosci. Res..

[bib23] Calias P. (2017). 2-Hydroxypropyl-β-cyclodextrins and the blood-brain barrier: considerations for Niemann-Pick disease type C1. Curr. Pharm. Des..

[bib24] Cermak R., Lupke G., Wolffram M., Breves S. (2006). In vitro degradation of the flavonol quercetin and of quercetin glycosides in the porcine hindgut. Arch. Anim. Nutr..

[bib25] Chabane M.N., Ahmad A.A., Peluso J., Muller C.D., Ubeaud G. (2009). Quercetin and naringenin transport across human intestinal Caco-2 cells. J. Pharm. Pharmacol..

[bib26] Chang L., Karin M. (2001). Mammalian MAP kinase signalling cascades. Nature.

[bib27] Chen C.Y., Kao C.L., Liu C.M. (2018). The cancer prevention, anti-inflammatory and anti-oxidation of bioactive phytochemicals targeting the TLR4 signaling pathway. Int. J. Mol. Sci..

[bib28] Chen L.L., Song C., Zhang Y., Li Y., Zhao Y.H., Lin F.Y., Han D.D., Dai M.H., Li W., Pan P.H. (2022). Quercetin protects against LPS-induced lung injury in mice via SIRT1-mediated suppression of PKM2 nuclear accumulation. Eur. J. Pharmacol..

[bib29] Cheng S.C., Wu Y.H., Huang W.C., Pang J.S., Huang T.H., Cheng C.Y. (2019). Anti-inflammatory property of quercetin through down regulation of ICAM-1 and MMP-9 in TNF-α-activated retinal pigment epithelial cells. Cytokine.

[bib30] Chessa M., Caddeo C., Valenti D., Manconi M., Sinico C., Fadda A.M. (2011). Effect of penetration enhancer containing vesicles on the percutaneous delivery of quercetin through new born pig skin. Pharmaceutics.

[bib31] Chitkara D., Nikalaje S.K., Mittal A., Chand M., Kumar N. (2012). Development of quercetin nanoformulation and in vivo evaluation using streptozotocin induced diabetic rat model. Drug Deliv. Transl. Res..

[bib32] Choy K.W., Murugan D., Leong X.F., Abas R., Alias A., Mustafa M.R. (2019). Flavonoids as natural anti-inflammatory agents targeting nuclear factor-kappa B (NFκB) signaling in cardiovascular diseases: a mini review. Front. Pharmacol..

[bib33] Costa A.C.D.F., de Sousa L.M., dos Santos Alves J.M., Goes P., Pereira K.M.A., Alves A.P.N.N., Vale M.L., Gondim D.V. (2021). Anti-inflammatory and hepatoprotective effects of quercetin in an experimental model of rheumatoid arthritis. Inflammation.

[bib34] Crespy V., Morand C., Besson C., Manach C., Demigne C., Remesy C. (2002). Quercetin, but not its glycosides, is absorbed from the rat stomach. J. Agric. Food Chem..

[bib35] Cui Z., Zhao X., Amevor F.K., Du X., Wang Y., Li D., Shu G., Tian Y., Zhao X. (2022). Therapeutic application of quercetin in aging-related diseases: SIRT1 as a potential mechanism. Front. Immunol..

[bib36] Dabeek W.M., Marra M.V. (2019). Dietary quercetin and kaempferol: bioavailability and potential cardiovascular-related bioactivity in humans. Nutrients.

[bib37] Dechsupa N., Kosintarajit P., Kamkan K., Khanjina T., Sirikul C., Innuan P., Suwan A., Anukul N., Kantapan J. (2022). Iron(III)-Quercetin complexes' safety for MRI cell tracking in cell therapy applications: cytotoxic and genotoxic assessment. Nanomaterials.

[bib38] Deepika, Maurya P.K. (2022). Health benefits of quercetin in age-related diseases. Molecules.

[bib39] Dehkordi E.A., Soveyzi F., Arian A.S., Hamedanchi N.F., Dehkordi A.H., Kopaei M.R. (2023). Quercetin and its role in reducing the expression of pro-inflammatory cytokines in osteoarthritis, Antiinflamm. Antiallergy Agents Med. Chem..

[bib40] DeMarino C., Schwab A., Pleet M., Mathiesen A., Friedman J., El-Hage N., Kashanchi F. (2017). Biodegradable nanoparticles for delivery of therapeutics in CNS infection. J. Neuroimmune Pharmacol..

[bib41] Dhawan S., Kapil R., Singh B. (2011). Formulation development and systematic optimization of solid lipid nanoparticles of quercetin for improved brain delivery. J. Pharm. Pharmacol..

[bib42] Dong Y., Wu X., Chen X., Zhou P., Xu F., Liang W. (2021). Nanotechnology shaping stem cell therapy: recent advances, application, challenges, and future outlook. Biomed. Pharmacother..

[bib43] Dreisietel A., Oosterhuis B., Vukman K.V., Schreier P., Oehme A., Locher S., Hajak G., Sand P.G. (2009). Berry anthocyanins and anthocyanidis exhibit distinct affinities for the efflux transporters BCRP and MDR1. Br. J. Pharmacol..

[bib44] Ebrahimpour S., Esmaelli A., Dehghanian F., Beheshti S. (2020). Effects of quercetin-conjugated with superparamagnetic iron oxide nanoparticles on learning and memory improvement through targeting microRNAs/NF-kB pathway. Sci. Rep..

[bib45] El-Fattah A.I.A., Fathy M.M., Ali Z.Y., El-Garawany A.E.R.A., Mohamed E.K. (2017). Enhanced therapeutic benefit of quercetin-loaded phytosome nanoparticles in ovariectomized rats. Chem. Biol. Interact..

[bib46] Endale M., Park S.C., Kim S., Kim S.H., Yang Y., Cho J.Y., Rhee M.H. (2013). Quercetin disrupts tyrosine-phosphorylated phosphatidylinositol 3-kinase and Myeloid differentiation factor-88 association, and inhibits MAPK/AP-1 and IKK/NF-κB-induced inflammatory mediators production in RAW 264.7 cells. Immunobiology.

[bib47] Fan H., Tang H.B., Shan L.Q., Liu S.C., Huang D.G., Chen X., Chen Z., Yang M., Yin X.H., Yang H., Hao D.J. (2019). Quercetin prevents necroptosis of oligodendrocytes by inhibiting macrophages/microglia polarization to M1 phenotype after spinal cord injury in rats. J. Neuroinflammation.

[bib48] Fasolo D., Bassani V.L., Teixeira H.F. (2009). Development of topical nanoemulsions containing quercetin and 3-O-methyquercetin. Pharmazie.

[bib49] Ferenczyova K., Kalocayova B., Bartekova M. (2020). Potential implications of quercetin and its derivatives in cardioprotection. Int. J. Mol. Sci..

[bib50] Fukaura M., Ishitsuka Y., Shirakawa S., Ushihama N., Yamada Y., Kondo Y., Takeo T., Nakagata N., Motoyama K., Higashi T., Arima H., Kurauchi Y., Seki T., Katsuki H., Higaki K., Matsuo M., Irie T. (2021). Intracerebroventricular treatment with 2-Hydroxypropyl-β-Cyclodextrin decreased cerebellar and hepatic glycoprotein nonmetastatic melanoma protein B (GPNMB) expression in Niemann-Pick Disease Type C model mice. Int. J. Mol. Sci..

[bib51] García-Mediavilla V., Crespo I., Collado P.S., Esteller A., Sánchez-Campos S., Tuñón M.J., González-Gallego J. (2007). The anti-inflammatory flavones quercetin and kaempferol cause inhibition of inducible nitric oxide synthase, cyclooxygenase-2 and reactive C-protein, and down-regulation of the nuclear factor kappaB pathway in Chang Liver cells. Eur. J. Pharmacol..

[bib52] Ge C., Wang S., Wu X., Lei L. (2023). Quercetin mitigates depression-like behavior via the suppression of neuroinflammation and oxidative damage in corticosterone-induced mice. J. Chem. Neuroanat..

[bib53] Ge Z., Xu M., Ge Y., Huang G., Chen D., Ye X., Xiao Y., Zhu H., Yin R., Shen H., Ma G., Qi L., Wei G., Li D., Wei S., Zhu M., Ma H., Shi Z., Wang X., Ge X., Qian X. (2023). Inhibiting G6PD by quercetin promotes degradation of EGFR T790M mutation. Cell Rep..

[bib54] Ghadi Z.S., Ebrahimnejad P., Amiri F.T., Nokhodchi A. (2021). Improved oral delivery of quercetin with hyaluronic acid containing niosomes as a promising formulation. J. Drug Target..

[bib55] Ghaffari F., Moghaddam A.H., Zare M. (2018). Neuroprotective effect of quercetin nanocrystal in a 6-Hydroxydopamine model of Parkinson disease: biochemical and behavioural evidence. Basic Clin. Neurosci..

[bib56] Ghosh A., Sarkar S., Mandal A.K., Das N. (2013). Neuroprotective role of nanoencapsulated quercetin in combating ischemia-reperfusion induced neuronal damage in young and aged rats. PLoS One.

[bib57] Ghosh S., Sarkar S., Choudhury S.T., Ghosh T., Das N. (2017). Triphenyl phosphonium coated nano-quercetin for oral delivery: neuroprotective effects in attenuating age related global moderate cerebral ischemia reperfusion injury in rats. Nanomedicine.

[bib58] Grewal A.K., Singh T.G., Sharma D., Sharma V., Singh M., Rahman M.H., Najda A., Janusz M.W., Kamel M., Albadrani G.M., Akhtar M.F., Saleem A., Daim M.M.A. (2021). Mechanistic insights and perspectives involved in neuroprotective action of quercetin. Biomed. Pharmacother..

[bib59] Guardia T., Rotelli A.E., Juarez A.O., Pelzer L.E. (2001). Anti-inflammatory properties of plant flavonoids. Effects of rutin, quercetin and hesperidin on adjuvant arthritis in rat. Il Farmaco.

[bib60] Haddad J.J. (2002). Cytokines and related receptor-mediated signaling pathways. Biochem Bioph Res Co.

[bib61] Hai Y., Zhang Y., Liang Y., Ma X., Qi X., Xiao J., Xue W., Luo Y., Yue T. (2020). Advance on the absorption, metabolism and efficacy exertion of quercetin and its important derivatives. Food Frontiers.

[bib62] Haribar U., Ulrih N.P. (2014). The metabolism of anthocyanins. Curr. Drug Metabol..

[bib63] Hatahet T., Morille M., Hommoss A., Devoisselle J.M., Muller R.H., Begu S. (2016). Quercetin topical application, from conventional dosage forms to nanodosage forms. Eur. J. Pharm. Biopharm..

[bib64] He X., Sun Y., Lu X., Yang F., Li T., Deng C., Song J., Huang X.A. (2023). Assessment of the anti‐inflammatory mechanism of quercetin 3, 7‐dirhamnoside using an integrated pharmacology strategy. Chem. Biol. Drug Des..

[bib65] Hisanaga A., Mukai R., Sakao K., Terao J., Hou D.X. (2016). Anti‐inflammatory effects and molecular mechanisms of 8‐prenyl quercetin. Mol. Nutr. Food Res..

[bib66] Hoffmann U., Kroemer H.K. (2004). The ABC transporters MDR1 and MRP2: multiple functions in disposition of xenobiotics and drug resistance. Drug Metab. Rev..

[bib67] Hossain M.K., Dayem A.A., Han J., Yin Y., Kim K., Saha S.K., Yang G.M., Choi H.Y., Cho S.G. (2016). Molecular mechanisms of the anti-obesity and anti-diabetic properties of flavonoids. Int. J. Mol. Sci..

[bib68] Hosseini A., Razavi B.M., Banach M., Hosseinzadeh H. (2021). Quercetin and metabolic syndrome: a review. Phytother Res..

[bib69] Hou D.D., Zhang W., Gao Y.L., Sun Y.Z., Wang H.X., Qi R.Q., Chen H.D., Gao X.H. (2019). Anti-inflammatory effects of quercetin in a mouse model of MC903-induced atopic dermatitis. Int. Immunopharmacol..

[bib70] Hou D.D., Zhang W., Gao Y.L., Sun Y.Z., Wang H.X., Qi R.Q., Chen H.D., Gao X.H. (2019). Anti-inflammatory effects of quercetin in a mouse model of MC903-induced atopic dermatitis. Int. Immunopharmacol..

[bib71] Hsieh H.L., Yu M.C., Cheng L.C., Chu M.Y., Huang T.H., Yeh T.S., Tsai M.M. (2022). Quercetin exerts anti-inflammatory effects *via* inhibiting tumor necrosis factor-Α-induced matrix metalloproteinase-9 expression in normal human gastric epithelial cells. World J. Gastroenterol..

[bib72] Huang K.T., Wu C.T., Chang Y., Ho F.M., Chiang C.K., Liu S.H. (2022). Therapeutic effect of quercetin polymeric nanoparticles on ischemia/reperfusion-induced acute kidney injury in mice. Biochem. Biophys. Res. Commun..

[bib73] Jafarinia M., Hosseini M.S., Kasiri N., Fazel N., Fathi F., Hakemi M.G., Eskandari N. (2020). Quercetin with the potential effect on allergic diseases. Allergy Asthma Clin. Immunol..

[bib74] Jia X., Gu M., Dai J., Wang J., Zhang Y., Pang Z. (2024). Quercetin attenuates *Pseudomonas aeruginosa*-induced acute lung inflammation by inhibiting PI3K/AKT/NF-κB signaling pathway. Inflammopharmacology.

[bib75] Joshi H., Gupta D.S., Kaur G., Singh T., Ramniwas S., Sak K., Aggarwal D., Chhabra R.S., Gupta M., Saini A.K., Tuli H.S. (2023). Nanoformulations of quercetin for controlled delivery: a review of preclinical anticancer studies. N. Schmied. Arch. Pharmacol..

[bib76] Kawaguchi K., Kaneko M., Miyake R., Takimoto H., Kumazawa Y. (2019). Potent inhibitory effects of quercetin on inflammatory responses of collagen-induced arthritis in mice. EMIDDT..

[bib77] Kumar P., Sharma G., Kumar R., Singh B., Malik R., Katare O.P., Raza K. (2016). Promises of a biocompatible nanocarrier in improved brain delivery of quercetin: biochemical, pharmacokinetic and biodistribution evidences. Int. J. Pharm..

[bib78] Kuo Y.C., Chen I.Y., Rajesh R. (2018). Use of functionalized liposomes loaded with antioxidants to permeate the blood-brain-barrier and inhibit β-amyloid-induced neurodegeneration in the brain. J. Taiwan Inst. Chem. Eng..

[bib79] Lee Y.J., Park Y. (2020). Green synthetic nanoarchitectonics of gold and silver nanoparticles prepared using quercetin and their cytotoxicity and catalytic applications. J. Nanosci. Nanotechnol..

[bib80] Li Y., Yao J., Han C., Yang J., Chaudhry M.T., Wang S., Liu H., Yin Y. (2016). Quercetin, inflammation and immunity. Nutrients.

[bib81] Liu H., Xue J.X., Li X., Ao R., Lu Y. (2013). Quercetin liposomes protect against radiation-induced pulmonary injury in a murine model. Oncol. Lett..

[bib82] Liu C., Liu D.Q., Tian Y.K., Mei W., Tian X.B., Xu A.J., Zhou Y.Q. (2022). The emerging role of quercetin in the treatment of chronic pain. Curr. Neuropharmacol..

[bib83] Lu Y., Yang J., Wang X., Ma Z., Li S., Liu Z., Fan X. (2020). Research progress in use of traditional Chinese medicine for treatment of spinal cord injury. Biomed. Pharmacother..

[bib84] Lu Z., Wang H., Ishfaq M., Han Y., Zhang X., Li X., Wang B., Lu X., Gao B. (2023). Quercetin and AMPK: a dynamic duo in alleviating MG-induced inflammation via the AMPK/SIRT1/NF-κB pathway. Molecules.

[bib85] Luan J., Zhu Y., Lin J., Zhang Y., Xu Q., Zhan L., Tian X., Zhao G., Peng X. (2023). Quercetin protects against *Aspergillus fumigatus* keratitis by reducing fungal load and inhibiting TLR-4 induced inflammatory response. Cytokine.

[bib86] Luo X., Bao X., Weng X., Bai X., Feng Y., Huang J., Liu S., Jia H., Yu B. (2022). The protective effect of quercetin on macrophage pyroptosis via TLR2/Myd88/NF-Κ and ROS/AMPK pathway. Life Sci..

[bib87] Maddan K., Lather V., Pandita D. (2016). Evaluation of polyamidoamine dendrimers as potential carriers for quercetin, a versatile flavonoid. Drug Deliv..

[bib88] Mamani-Matsuda M., Kauss T., Al-Kharrat A., Rambert J., Fawaz F., Thiolat D., Moynet D., Coves S., Malvy D., Mossalayi M.D. (2006). Therapeutic and preventive properties of quercetin in experimental arthritis correlate with decreased macrophage inflammatory mediators. Biochem. Pharmacol..

[bib89] Mariano A., Bigioni I., Marchetti M., d'Abusco A.S., Superti F. (2023). Repositioned natural compounds and nanoformulations: a promising combination to counteract cell damage and inflammation in respiratory viral infections. Molecules.

[bib90] Moradi S.Z., Momtaz S., Bayrami Z., Farzaei M.H., Abdollahi M. (2020). Nanoformulations of herbal extracts in treatment of neurodegenerative disorders. Front. Bioeng. Biotechnol..

[bib91] Mukhopadhyay P., Maity S., Mandal S., Chakraborti A.S., Prajapati A.K., Kundu P.P. (2018). Preparation, characterization and in vivo evaluation of pH sensitive, safe quercetin-succinylated chitosan-alginate core-shell-corona nanoparticle for diabetes treatment. Carbohydr. Polym..

[bib92] Muresan M., Olteanu D., Filip G.A., Clichici S., Baldea I., Jurca T. (2021). Comparative study of the pharmacological properties and biological effects of Polygonum aviculare L. Herba extract-entrapped liposomes versus quercetin-entrapped liposomes on doxorubicin-induced toxicity on HUVECs. Pharmaceutics.

[bib93] Naser S.S., Singh D., Preetam S., Kishore S., Kumar L., Nandi A., Simnani F.Z., Choudhury A., Sinha A., Mishra Y.K., Suar M., Panda P.K., Malik S., Verma S.K. (2023). Posterity of nanoscience as lipid nanosystems for Alzheimer's disease regression. Mater. Today Bio..

[bib94] Nemeth K., Plumb G.W., Berrin J.G., Juge N., Jacob R., Naim H.Y., Williamson G., Swallow D.M., Kroon P.A. (2003). Deglycosylation by small intestinal epithelial cell beta-glucosidases is a critical step in the absorption and metabolism of dietary flavonoid glycosides in humans. Eur. J. Nutr..

[bib95] Neufurth M., Wang X., Wang S., Schroder H.C., Muller W.E.G. (2021). Caged dexamethasone/quercetin nanoparticles, formed of the morphogenetic active inorganic polyphosphate, are strong inducers of MUC5AC. Mar. Drugs.

[bib96] Panche A.N., Diwan A.D., Chandra S.R. (2016). Flavonoids: an overview. J. Nutr. Sci..

[bib97] Patel H.S., Shaikh S.J., Ray D., Aswal V.K., Vaidya F., Pathak C., Sharma R.K. (2022). Formulation, solubilization, and in vitro characterization of quercetin-incorporated mixed micelles of PEO-PPO-PEO block copolymers. Appl. Biochem. Biotechnol..

[bib98] Pei J., Kumarasamy R.V., Jayaraman S., Kanniappan G.V., Long Q., Palanisamy C.P. (2025). Quercetin-functionalized nanomaterials: innovative therapeutic avenues for Alzheimer's disease management. Ageing Res. Rev..

[bib99] Petri N., Tannergren C., Holst B., Mellon F.A., Bao Y., Plumb G.W., Bacon J., O'Leary K.A., Kroon P.A., Knutson L., Forsell P., Eriksson T., Lennernas H., Williamson G. (2003). Absorption/metabolism of sulphoraphane and quercetin and regulation of phase II enzymes in human jejunum in vivo. Drug Metab. Dispos..

[bib100] Pierro F.D., Iqtadar S., Khan A., Mumtaz S.U., Chaudhry M.M., Bertuccioli A., Derosa G., Maffioli P., Togni S., Riva A., Allegrini P., Khan S. (2021). Potential clinical benefits of quercetin in the early stage of COVID-19: results of a second, pilot, randomized, controlled and open-label clinical trial. Int. J. Gen. Med..

[bib101] Pooviah N., Davoudi Z., Peng H., Schlichtmann B., Mallapragada S., Narasimhan B., Wang Q. (2018). Treatment of neurodegenerative disorders through the blood-brain barrier using nanocarriers. Nanoscale.

[bib102] Ramesh P., Jagadeesan R., Sekaran S., Dhanasekaran A., Vimalraj S. (2021). Flavonoids: classification, function and molecular mechanisms involved in bone remodelling. Front. Endocrinol..

[bib103] Riemschneider S., Hoffmann M., Slanina U., Weber K., Hauschildt S., Lehmann J. (2021). Indol-3-Carbinol and quercetin ameliorate chronic DSS-induced colitis in C57bl/6 mice by AhR-mediated anti-inflammatory mechanisms. Int. J. Environ. Res. Publ. Health.

[bib104] Rio D.D., Mateos A.R., Spencer J.P.E., Tognolini M., Borges G., Crozier A. (2013). Dietary (poly) phenolics in human health: structures, bioavailability and evidence of protective effects against chronic diseases, Antioxid. Redox Signal.

[bib105] Riva A., Ronchi M., Petrangolini G., Bosisio S., Allegrini P. (2019). Improved oral absorption of quercetin from quercetin phytosome, a new delivery system based on food grade lecithin. Eur. J. Drug Metab. Pharmacokinet..

[bib106] Rivera L., Moron R., Sanchez M., Zarzuelo A., Galisteo M. (2008). Quercetin ameliorates metabolic syndrome and improves the inflammatory status in obese Zucker rats. Obesity.

[bib107] Rossol M., Heine H., Meusch U., Quandt D., Klein C., Sweet M.J., Hauschildt S. (2011). LPS-induced cytokine production in human monocytes and macrophages. Crit. Rev. Immunol..

[bib108] Russo M., Spagnuolo C., Tedesco I., Bilotto S., Russo G.L. (2012). The flavonoid quercetin in disease prevention and therapy: facts and fancies. Biochem. Pharmacol..

[bib109] Santos E.L., Maia B.H.L.N.S., Ferriani A.P., Teixeira S.D. (2017). Flavonoids: classification, biosynthesis and chemical ecology. Flavonoids - From Biosynthesis to Human Health.

[bib110] Sapino S., Ugazio E., Gastaldi L., Miletto I., Berlier G., Zonari D., Bosso S.O. (2015). Mesoporous silica as topical nanocarriers for quercetin: characterization and in vitro studies. Eur. J. Pharm. Biopharm..

[bib111] Sarrias A.G., Villalba R.G., Vaquero M.R., Alasalvar C., Orem A., Zafrilla P., Barberan F.A.T., Selma M.V., Espin J.C. (2017). Clustering according to urolithin metabotype explains the interindividual variability in the improvement of cardiovascular risk biomarkers in overweight-obese individuals consuming pomegranate: a randomized clinical trial. Mol. Nutr. Food Res..

[bib112] Scalia S., Franceschinis E., Bertelli D., Iannuccelli V. (2013). Comparative evaluation of the effect of permeation enhancers, lipid nanoparticles and colloidal silica on in vivo human skin penetration of quercetin. Skin Pharmacol. Physiol..

[bib113] Schwingel T.E., Klein C.P., Nicoletti N.F., Dora C.L., Hadrich G., Bica C.G., Lopes T.G., da Silva V.D., Morrone F.B. (2014). Effects of the compounds resveratrol, rutin, quercetin, and quercetin nanoemulsion on oxaliplatin-induced hepatotoxicity and neurotoxicity in mice. Naunyn-Schmiedebergs Arch Pharmacol.

[bib114] Sesink A.L.A., Arts I.C.W., de Boer V.C.J., Breedveld P., Schellens J.H.M., Hollman P.C.H., Russel F.G.M. (2005). Breast cancer resistance protein (BCRP1/ABCG2) limits net intestinal uptake of quercetin in rats by facilitating apical efflux of glucuronides. Mol. Pharmacol..

[bib115] Shabbir U., Rubab M., Daliri E.B.M., Chelliah R., Javed A., Oh D.H. (2021). Curcumin, quercetin, catechins and metabolic diseases: the role of gut microbiota. Nutrients.

[bib116] Shanmugasundaram D., Roza J.M. (2022). Assessment of anti-inflammatory and antioxidant activity of quercetin-rutin blend (SophorOx™) - an *in-vitro* cell based assay. JCIM.

[bib117] Silva M.F., Silva C.F., Carvalheiro M.C., Simoes S., Marinho H.S., Marcelino P., Campos M.C., Metselaar J.M., Fernandes E., Baptista P.V., Farnandes A.R., Corvo M.L. (2022). Quercetin liposomal nanoformulation for Ischemia and Reperfusion injury treatment. Pharmaceutics.

[bib118] Song Y.K., Yoon J.H., Woo J.K., Kang J.H., Lee K.R., Oh S.H., Chung S.J., Maeng H.J. (2020). Quercetin is a flavonoid breast cancer resistance protein inhibitor with an impact on the oral pharmacokinetics of sulfasalazine in rats. Pharmaceutics.

[bib119] Spagnuolo C., Moccia S., Russo G.L. (2018). Anti-inflammatory effects of flavonoids in neurodegenerative disorders. Eur. J. Med. Chem..

[bib120] Sun H., Zhan M., Zou Y., Ma J., Liang J., Tang G., Laurent R., Mignani S., Majoral J.P., Shi X., Shen M. (2025). Bioactive phosphorus dendrimers deliver protein/drug to tackle osteoarthritis via cooperative macrophage reprogramming. Biomaterials.

[bib121] Taghipour Y.D., Hajialyani M., Naseri R., Hesari M., Mohammadi P., Stefanucci A., Mollica A., Farzaei M.H., Abdollahi M. (2019). Nanoformulations of natural products for management of metabolic syndrome. Int. J. Nanomed..

[bib122] Tang J., Diao P., Shu X., Li L., Xiong L. (2019). Quercetin and quercitrin attenuates the inflammatory response and oxidative stress in LPS-induced RAW264.7 cells: *in vitro* assessment and a theoretical model. BioMed Res. Int..

[bib123] Terao J. (2017). Factors modulating bioavailability of quercetin-related flavonoids and the consequences of their vascular function. Biochem. Pharmacol..

[bib124] Testa G., Gamba P., Badilli U., Gargiulo S., Maina M., Guina T., Calfapietra S., Biasi F., Cavalli R., Poli G., Leonarduzzi G. (2014). Loading into nanoparticles improves quercetin's efficacy in preventing neuroinflammation induced by oxysterols. PLoS One.

[bib125] Tomou E.M., Papakyriakopoulou P., Saitani E.M., Valsami G., Pippa N., Skaltsa H. (2023). Recent advances in nanoformulations for quercetin delivery. Pharmaceutics.

[bib126] Tomou E.M., Papakyriakopoulou P., Saitani E.M., Valsami G., Pippa N., Skaltsa H. (2023). Recent advances in nanoformulations for quercetin delivery. Pharmaceutics.

[bib127] Tong F., Liu S., Yan B., Li X., Ruan S., Yang S. (2017). Quercetin nanoparticle complex attenuated diabetic nephropathy via regulating the expression level of ICAM-1 on endothelium. Int. J. Nanomed..

[bib128] Ulusoy H.G., Sanlier N. (2020). A minireview of quercetin: from its metabolism to possible mechanisms of its biological activities. Crit. Rev. Food Sci. Nutr..

[bib129] Vian C.D.O., Marinho M.A.G., Marques M.D.S., Hort M.A., Cordeiro M.F., Horn A.P. (2024). Effects of quercetin in preclinical models of Parkinson's disease: a systematic review. Basic Clin. Pharmacol. Toxicol..

[bib131] Vishwas S., Kumar R., Khursheed R., Ramanunny A.K., Kumar R., Awasthi A., Corrie L., Porwal O., Arshad M.F., Alshammari M.K., Alghitran A.A., Qumayri A.N., Alkhaldi S.M., Alshammari A.K., Chellappan D.K., Gupta G., Collet T., Adams J., Dua K., Gulati M., Singh S.K. (2023). Expanding arsenal against neurodegenerative diseases using quercetin based nanoformulations: breakthroughs and bottlenecks. Curr. Neuropharmacol..

[bib132] Wang N., Li F., Du J., Hao J., Wang X., Hou Y., Luo Z. (2024). Quercetin protects against global cerebral ischemia‒reperfusion injury by inhibiting microglial activation and polarization. J. Inflamm. Res..

[bib133] Williamson G., Kay C.D., Crozier A. (2018). The bioavailability, transport and bioactivity of dietary flavonoids: a review from a historical perspective. Compr. Rev. Food Sci. Food Saf..

[bib134] Wolffram S., Block M., Ader P. (2002). Quercetin-3-glucoside is transported by the glucose carrier SGLT1 across the brush border membrane of rat small intestine. J. Nutr..

[bib135] Xiao X., Shi D., Liu L., Wang J., Xie X., Kang T., Deng W. (2011). Quercetin suppresses cyclooxygenase-2 expression and angiogenesis through inactivation of P300 signaling. PLoS One.

[bib136] Xie J., Shen Z., Anraku Y., Kataoka K., Chen X. (2019). Nanomaterial-based blood-brain-barrier (BBB) crossing strategies. Biomaterials.

[bib137] Xu W., Xie S., Chen X., Pan S., Qian H., Zhu X. (2021). Effects of quercetin on the efficacy of various chemotherapeutic drugs in cervical cancer cells. Drug Des. Dev. Ther..

[bib138] Yang W.S., Jeong D., Yi Y.S., Lee B.H., Kim T.W., Htwe K.M., Kim Y.D., Yoon K.D., Hong S., Lee W.S., Cho J.Y. (2014). Myrsine seguinii ethanolic extract and its active component quercetin inhibit macrophage activation and peritonitis induced by LPS by targeting to Syk/Src/IRAK-1. J. Ethnopharmacol..

[bib139] Yarjanli Z., Ghaedi K., Esmaelli A., Zarrabi A., Rahgozar S. (2019). The antitoxic effects of quercetin and quercetin-conjugated iron oxide nanoparticles (QNPs) against H2O2-induced toxicity in PC12 cells. Int. J. Nanomed..

[bib140] Yuan Z.P., Chen L.J., Fan L.Y., Tang M.H., Yang G.L., Yang H.S. (2006). Liposomal quercetin efficiently suppresses growth of solid tumours in murine models. Clin. Cancer Res..

[bib141] Zang X., Cheng M., Zhang X., Chen X. (2021). Quercetin nanoformulations: a promising strategy for tumor therapy. Food Funct..

[bib142] Zhang L., Zuo Z., Lin G. (2007). Intestinal and hepatic glucuronidation of flavonoids. Mol. Pharm..

[bib143] Zhang Y., Qu X., Gao H., Zhai J., Tao L., Sun J., Song Y., Zhang J. (2020). Quercetin attenuates NLRP3 inflammasome activation and apoptosis to protect INH-induced liver injury via regulating SIRT1 pathway. Int. Immunopharmacol..

[bib144] Zhang Y., Guan R., Huang H. (2023). Anti-allergic effects of quercetin and quercetin liposomes in RBL-2H3 cells. Endocr., Metab. Immune Disord.: Drug Targets.

[bib145] Zhao X., Wang J., Deng Y., Liao L., Zhao M., Peng C., Li Y. (2021). Quercetin as a protective agent for liver diseases: a comprehensive descriptive review of the molecular mechanism. Phytother Res..

